# **Role of Nasal Skin Massage in Optimizing Secondary Rhinoplasty Results**

**Published:** 2016-01

**Authors:** Ali Manafi, Farzad Manafi

**Affiliations:** 1 Department of Plastic Surgery, Iran University of Medical Sciences, Tehran, Iran;; 2 Anatomical Research Center, Iran University of Medical Sciences, Tehran, Iran

**Keywords:** Nose, Skin, Massage, Secondary rhinoplasty

Rhinoplasty is the most frequent aesthetic surgery in Iran.^1^ Recent reports signifies its prevalence rate as high as 180 cases of rhinoplasty per 100,000 population which is the highest in the world.^1^^,^^2^ In addition to plastic surgeons, there are many other surgeons performing rhinoplasty surgery without academic aesthetic surgery training. With increasing the rate of rhinoplasty by the academically untrained surgeons, chance of secondary deformities has been increased dramatically.

A pervasive trend among Iranian patients and surgeons is to miniaturize the nose as much as possible, which usually results in a pathologically small nose. After ceasing the edema, these patients develop a small malformed nose with evident breathing problems.^3^^-^^5^ So they start complaining about the shape, size and function of the nose. They wish to have an a esthetically pleasant nose with proportional size. 

Severe skin shortage makes secondary surgeries unsuccessful in most secondary rhinoplasty cases, especially in patients with foreshortened nose. It makes soft tissue dissection, skeletonization and graft implantation extremely laborious and sub-standardizes function as well as aesthetic results. Surgical attempt for inserting different types and shaped grafts especially large and extended grafts in these cases, will go to fail due to inability of tensionless skin closure.^6^^-^^13^

Since 15 years ago, we have implemented skin massage as a new approach in these patients. They have been trained for nasal skin massage technique with manipulating and putting traction on the nasal skin in forward and downward directions. These manipulations should be performed at all directions, mostly in forward and downward directions and all parts of the nose (Figure 1). Massage performed 6-8 times a day intermittently for 2-5 minutes (per each session) in 3 consecutive months. It has been recommended to do it bimanually or with help of two opposing fingers of dominant hand with application of some mineral or organic oil in order to lubricate and soften the skin.

**Fig. 1:**
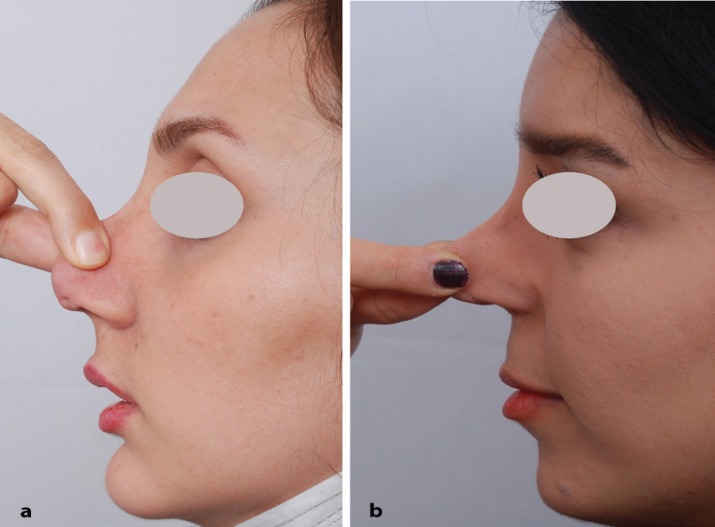
Directions and effectiveness of skin traction of nose in two difficult cases of tertiary rhinoplasty

 Patients will experience remarkable skin softening after several weeks of massage therapy. A follow-up visit after 12 weeks of massage therapy is recommended, in which the surgeon determines the proper plan based on the amount of skin laxity (Figure 2). In case of acceptable result, surgery could be performed, otherwise the patient is asked for applying another 3 months period of massage therapy.

**Fig. 2:**
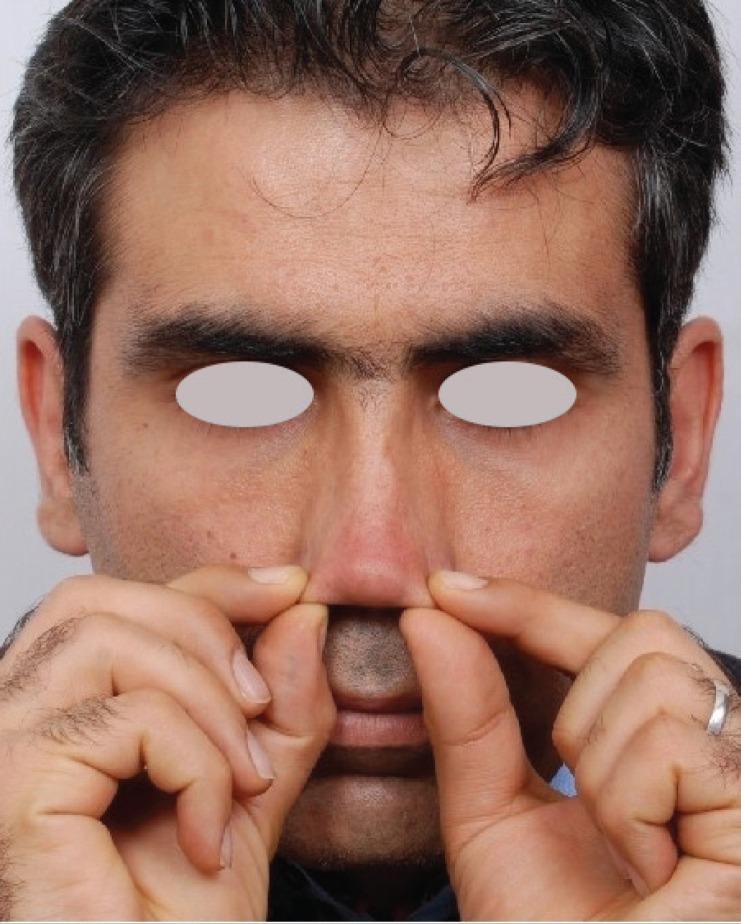
Results of effective skin massage in male patient

This approach has several advantages: First of all, skin would be degloved easily during the surgery and allows safe dissection. Intraoperative skin damage and tearing would be significantly reduced especially in those patients who have undergone multiple rhinoplasties. Secondly, it seems that blood supply of the skin has been increased dramatically due to continuous massage therapy and skin ischemia and necrosis are less frequently seen in these patients.

Third benefit is that, these patients are actively involved in treatment process and mentally prepared for surgery and post-operative care. Since 15 years ago, it has been our routine approach for secondary cases which comprise about 35% of our rhinoplasties. Effect of the skin massage in improvement of post-operative results has been demonstrated in the following photos (Figure 3 and 4).

**Fig. 3:**
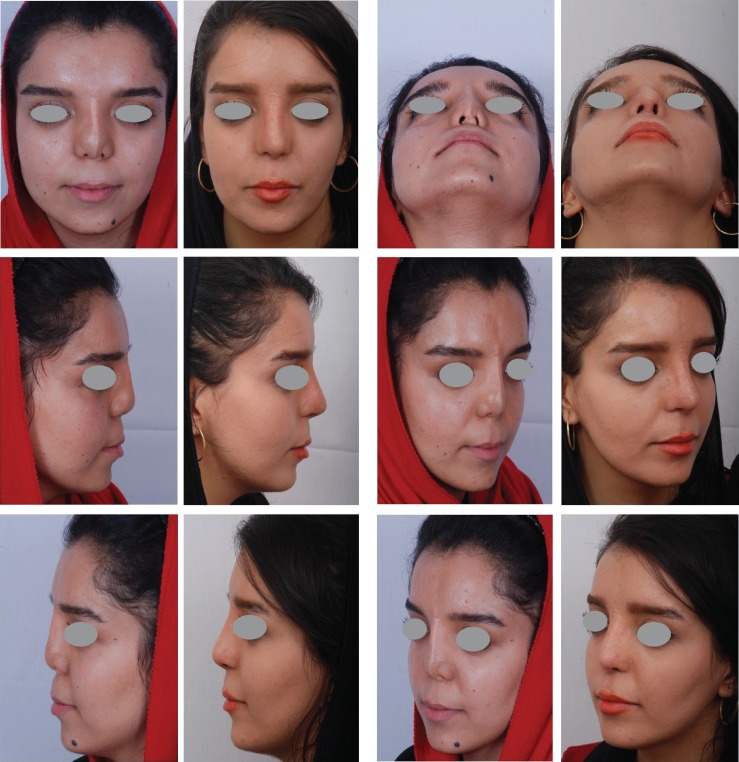
Case of ultra-short nose (Binder’s syndrome) with 3 previous operations in each pair of pictures the left one is pre-op and the right one is post-op. Results (8 months post-op) after nasal augmentation with grated cartilage + fascia from rib cartilage and rectus abdominis fascia and other different grafts for all parts of the nose. We have also used grated cartilage + fascia for augmentation of deficient nasal base and lower rim of piriform aperture

**Fig. 4:**
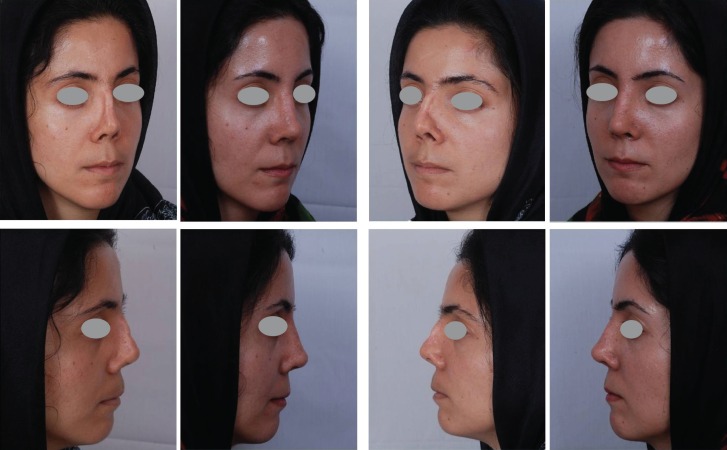
Deformed tertiary nose after 3 previous rhinoplasties, bone and cartilage graft. Result after 10 months: Lengthening and multiple grafting from autologous rib cartilage and grated cartilage graft + fascia

## CONFLICT OF INTEREST

The authors declare no conflict of interest.
